# Association of Functional *ADRB1* (rs1801252) and *ADRB2* (rs1042714) Polymorphisms with Primary Open-Angle Glaucoma in a Saudi Cohort

**DOI:** 10.3390/ijms27156668

**Published:** 2026-07-26

**Authors:** Altaf A. Kondkar, Tahira Sultan, Taif A. Azad, Essam A. Osman, Faisal A. Almobarak, Ehab Y. Alsirhy, Saleh A. Al-Obeidan

**Affiliations:** Glaucoma Research Chair, Department of Ophthalmology, College of Medicine, King Saud University, Riyadh 11411, Saudi Arabia

**Keywords:** primary open-angle glaucoma, β-adrenergic receptors, *ADRB1*, *ADRB2*, rs1801252, rs1042714, Ser49Gly, Gln27Glu, Saudi Arabia, genetic association, single nucleotide polymorphism

## Abstract

This case–control study investigated the association between β-adrenergic receptors, *ADRB1* rs1801252 (A-to-G/Ser49Gly) and *ADRB2* rs1042714 (C-to-G/Gln27Glu), polymorphisms and primary open-angle glaucoma (POAG) in a Saudi cohort. Genotyping of 418 participants (167 cases and 251 controls) was performed using real-time PCR assays. Analysis was performed among 412 participants with complete genotype data for both SNPs (163 cases and 249 controls). The *ADRB1* rs1801252-G (Gly49) allele was significantly associated with increased POAG risk across allelic (adjusted odds ratio (OR) = 1.93; 95% confidence interval (CI) = 1.22–3.08; *p* = 0.005), dominant (adjusted OR = 2.13; 95% CI = 1.23–3.70; *p* = 0.007), and additive (adjusted OR per G allele = 1.93; 95% CI = 1.22–3.08; *p* = 0.005) models, surviving Bonferroni correction (α = 0.025). In contrast, *ADRB2* rs1042714 showed no significant association under any model. Combined two-locus allelic analysis suggested a nominally increased risk for the C–G combined profile (*ADRB2*C + *ADRB1*G; OR = 1.91; *p* = 0.033). No significant genotype associations were observed with intraocular pressure or cup-to-disc ratio within the POAG cohort. These findings suggest that *ADRB1* rs1801252 may contribute to POAG susceptibility in this population; replication in larger cohorts and targeted functional follow-up studies are warranted to elucidate pressure-independent mechanisms.

## 1. Introduction

Primary open-angle glaucoma (POAG) is the most prevalent subtype of glaucoma, affecting approximately 3.07% of the population in Middle Eastern regions and representing the leading cause of irreversible blindness worldwide [1]. POAG is a multifactorial optic neuropathy characterized by progressive retinal ganglion cell loss, retinal nerve fiber layer thinning, optic disc cupping, and corresponding visual field defects [2]. Elevated intraocular pressure (IOP), largely resulting from impaired aqueous humor outflow, remains the most important modifiable risk factor for POAG onset and progression [3]. However, increasing evidence indicates that vascular dysregulation, oxidative stress, mitochondrial dysfunction, and impaired neuroprotection also contribute substantially to POAG pathogenesis alongside elevated IOP [2,4,5].

Genetic factors substantially contribute to POAG susceptibility. Multi-ancestry genome-wide studies and candidate-gene investigations have highlighted pathways that mediate trabecular meshwork function and ocular perfusion, such as extracellular matrix remodelling and vascular biology [6,7,8]. Despite these advances, the polygenic and heterogeneous nature of POAG underscores the value of targeting functionally relevant variants, particularly in the underrepresented Middle Eastern populations.

Adrenergic signaling is a biologically relevant pathway in glaucoma because β-adrenergic receptors regulate both aqueous humor dynamics and ocular vascular tone [9,10,11]. β-adrenergic receptors are expressed in the ciliary body, trabecular meshwork, and ocular vascular tissues. [12]. Among these, the *ADRB1* and *ADRB2* genes encode the β1- and β2-adrenergic receptors, respectively, and are considered important functional candidates in glaucoma biology because genetic variation at these loci can alter receptor expression, desensitization, intracellular signaling, and downstream physiological responses [13,14,15,16].

The *ADRB1* rs1801252 (A-to-G, Ser49Gly) polymorphism is a functional non-synonymous variant located in the N-terminal extracellular domain of the receptor. This variant influences receptor downregulation and desensitization, with the Gly49 isoform demonstrating enhanced agonist-induced downregulation compared with the Ser49 form [17,18,19]. Such functional differences may alter adrenergic signaling efficiency in ocular tissues and thereby influence glaucoma susceptibility. Likewise, *ADRB2* rs1042714 (C-to-G, Gln27Glu) affects β2-receptor regulation and may modulate downstream signaling in a tissue-specific manner, but its role in ocular disease susceptibility remains unclear [20,21]. Previous studies investigating adrenergic receptor polymorphisms in glaucoma have yielded inconsistent findings across populations. In an Indian cohort, *ADRB2* rs1042714 GG (Glu27) and rs1042713 GA genotypes were associated with increased POAG risk [14]. In contrast, larger case–control studies in white and black African populations reported no significant association between *ADRB2* codon 16/27 variants and POAG susceptibility [22]. A Japanese study further showed that *ADRB1* Arg389Gly (rs1801253) and *ADRB2* variants influenced glaucoma-related clinical traits such as age at diagnosis and IOP, but not overall disease risk, except in normal-tension glaucoma [13]. These discrepancies suggest that the contribution of adrenergic receptor polymorphisms to glaucoma may be population-specific and influenced by ancestry-related genetic architecture, underscoring the need for population-specific studies in Middle Eastern cohorts.

To date, no study has examined *ADRB1* rs1801252 (Ser49Gly) gene located on Chromosome 10q25.3 and *ADRB2* rs1042714 (Gln27Glu) gene located on Chromosome 5q32 in relation to POAG in a Saudi or broader Middle Eastern population. Therefore, this study aimed to investigate the association of these functional polymorphisms with POAG susceptibility in a Saudi cohort, evaluate their relationship with clinical markers of disease severity, including IOP and cup-to-disc ratio (CDR), and assess the potential effects of combined allelic profiles on POAG risk.

## 2. Results

### 2.1. Study Population Characteristics and Quality Control

The study included a total of 418 participants: 167 POAG cases and 251 healthy controls. Demographic and clinical characteristics are presented in Table 1. The mean age of the POAG group was slightly higher than the control group (61.3 ± 10.5 vs. 59.9 ± 7.8 years; *p* = 0.046), while gender distribution was comparable between the two groups (*p* = 0.747, 56.3% vs. 54.2% male, respectively). Both rs1801252 and rs1042714 demonstrated an overall genotyping call rate of 98.6% and did not show significant deviation from Hardy–Weinberg Equilibrium (HWE) in the control group (*p* = 0.092 and *p* = 0.220, respectively).

### 2.2. Association of ADRB1 and ADRB2 Polymorphisms with POAG Risk

Genotype and allele frequencies of *ADRB1* (rs1801252) and *ADRB2* (rs1042714) were analyzed for association with POAG susceptibility under multiple genetic models (Table 2). After excluding samples with incomplete genotyping data for either SNP, the final analysis included 412 participants (163 POAG cases and 249 controls).

#### 2.2.1. *ADRB1* (rs1801252)

Statistical analysis revealed a significant and consistent association between the *ADRB1* rs1801252 (Ser49Gly) polymorphism and POAG susceptibility. In the allelic association analysis, the frequency of the minor G (Gly) allele was significantly higher in POAG cases compared to controls (14.1% vs. 6.8%; odds ratio (OR) = 2.24; 95% confidence interval (CI): 1.40–3.58; unadjusted *p* = 0.007, adjusted *p* = 0.005).

Genotype analysis further confirmed this risk association. The *ADRB1* rs1801252 polymorphism showed a significant association with POAG susceptibility under the global co-dominant model (unadjusted *p* = 0.011; adjusted *p* = 0.026) but did not survive Bonferroni correction. Furthermore, carriers of the G-allele had a 2.21-fold increased crude risk of POAG compared to A/A homozygotes (95% CI: 1.31–3.72) in the dominant model. Risk was most pronounced in the allelic model (adjusted *p* = 0.005) and the dominant model (adjusted *p* = 0.007). The additive trend model further confirmed a significant increase in risk for each additional copy of the G-allele (adjusted *p* = 0.005), demonstrating a clear dose–response relationship.

#### 2.2.2. *ADRB2* (rs1042714)

In contrast, the *ADRB2* rs1042714 (Gln27Glu) polymorphism did not show a statistically significant association with POAG risk in this Saudi cohort. The frequency of the minor G allele was comparable between cases and controls (25.5% vs. 23.3%; OR = 1.12; 95% CI: 0.81–1.56; unadjusted *p* = 0.486, adjusted *p* = 0.506). The global genotype distribution for *ADRB2* rs1042714 did not differ significantly between cases and controls (unadjusted *p* = 0.776; adjusted *p* = 0.691). No significant associations were observed for rs1042714 under any other genetic or adjusted models (all *p* > 0.05).

### 2.3. Multivariate Logistic Regression Analysis

**Figure 1:**
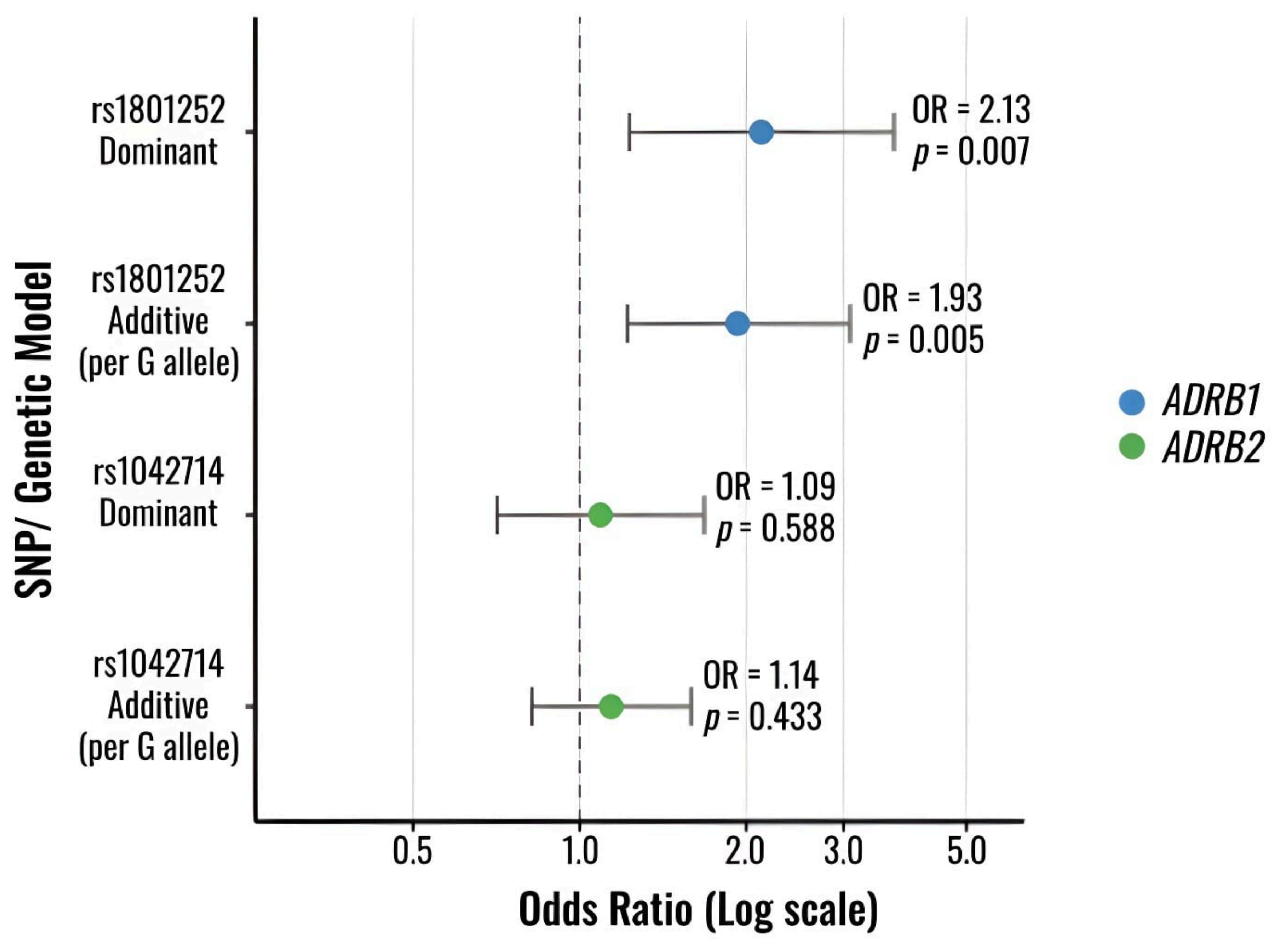
Forest plot of the association between rs1801252 (*ADRB1*) and rs1042714 (*ADRB2*) polymorphisms and POAG risk. The plot displays odds ratios (OR) represented by solid circles and 95% confidence intervals (CI) by horizontal bars. Results are shown for the Dominant and Additive models in covariate-adjusted (age and gender) formats. The dashed vertical line indicates the null effect (OR = 1.0). Specific OR and *p*-values are indicated. Associations are considered statistically significant where the 95% CI does not overlap the null line. rs1801252 (*ADRB1*) demonstrates consistent significance across both models (*p* < 0.05), whereas rs1042714 (*ADRB2*) estimates are non-significant with 95% CI consistently crossing the null line.

To verify the independence of these findings, a multivariate logistic regression analysis was performed, adjusting for age and gender as potential confounders. The independent association between *ADRB1* rs1801252 and POAG remained significant after adjustment in the dominant model (adjusted OR = 2.13; 95% CI: 1.23–3.70; *p* = 0.007) and the additive model (adjusted OR = 1.93; 95% CI: 1.22–3.08; *p* = 0.005), retaining significance after Bonferroni correction. No significant independent association was observed for *ADRB2* rs1042714 following multivariate adjustment. The Forest plot (Figure 1) visually demonstrates the associations across both SNPs and genetic models.

### 2.4. Combined Two-Locus Allelic Association

Linkage disequilibrium (LD) analysis between rs1042714 (C-to-G, *ADRB2*) and rs1801252 (A-to-G, *ADRB1*) situated on distinct chromosomes revealed an r^2^ value of 0.0005, confirming that these variants are not in LD and are inherited independently. Subsequently, a combined two-locus allelic frequency association analysis was performed to assess potential cumulative contributions of allelic combinations at both loci on POAG risk (Table 3).

The C-A (major-major allele profile) was observed in 72.1% of controls and 66.4% of POAG cases and used as the reference group. The C-G combined profile (comprising the *ADRB2* C-allele and the *ADRB1* G-allele) showed nominal significance and was more prevalent in POAG cases compared to controls (8.2% vs. 4.5%; OR = 1.91; 95% CI: 1.06–3.44; *p* = 0.033), suggesting a possible cumulative effect on disease susceptibility. The G-A allelic combination was non-significant. The G-G profile (double-minor combination) showed a higher frequency in cases (4.4% vs. 2.2%) but did not reach statistical significance (OR = 2.02, 95% CI: 0.90–4.55, *p* = 0.096), likely reflecting limited statistical power due to its low frequency in this population (Table 3).

### 2.5. Genotype-Phenotype Correlation in POAG Cases

**Figure 2:**
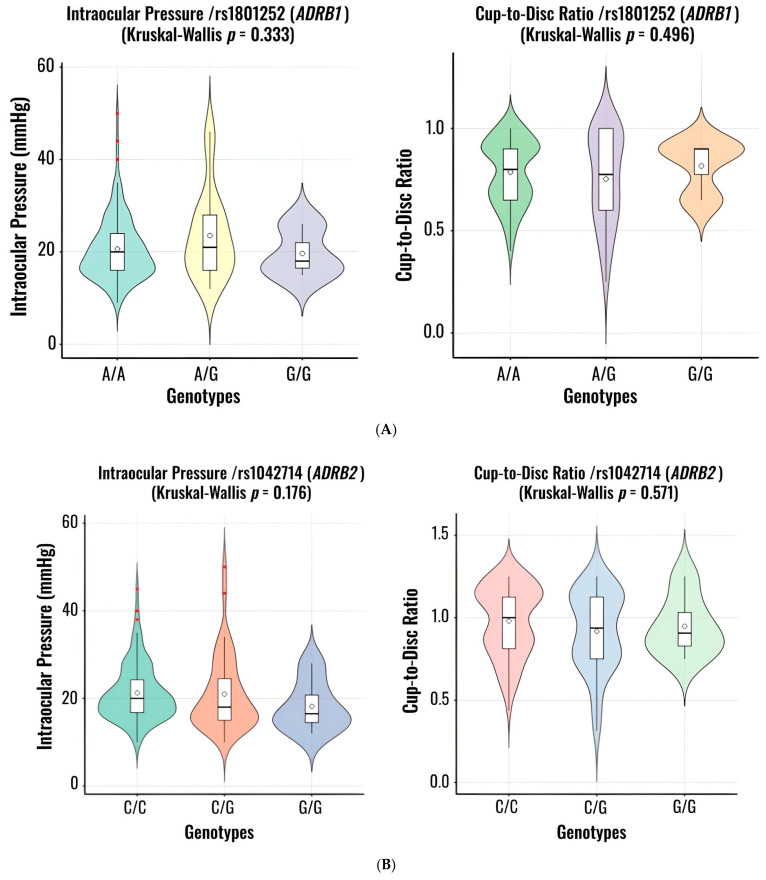
Violin plot representing comparison of clinical parameters across *ADRB1* and *ADRB2* genotypes in POAG patients. Distribution of (**A**) Intraocular Pressure (IOP) and Cup-to-Disc Ratio (CDR) according to *ADRB1* (rs1801252) genotypes; and (**B**) IOP and CDR according to *ADRB2* (rs1042714) genotypes. Statistical analysis revealed no significant association between these genetic variants and clinical markers of disease severity (all *p* > 0.05, Kruskal–Wallis H-test).

To evaluate whether the studied genetic variants influenced clinical severity of glaucoma, we analyzed the correlation between genotypes and key clinical parameters (IOP and CDR) within the POAG patient cohort. Importantly, all IOP values and CDRs used in these genotype–phenotype association analyses represent untreated baseline measurements, thereby eliminating potential confounding treatment effects. Prior to analysis, the distribution of clinical data was assessed using the Shapiro–Wilk test, which indicated that both IOP (*p* < 0.001) and CDR (*p* < 0.001) deviated significantly from normal distribution. Consequently, the non-parametric Kruskal–Wallis H-test was employed for all phenotypic comparisons (Figure 2). Furthermore, evaluation of IOP and CDR across combined two-locus genotype profiles revealed no significant associations (Kruskal–Wallis *p* = 0.299 for IOP and *p* = 0.532 for CDR; Supplementary Table S1).

## 3. Discussion

This study is the first to investigate the association of *ADRB1* rs1801252 (Ser49Gly) and *ADRB2* rs1042714 (Gln27Glu) polymorphisms with POAG in a Saudi Arabian cohort. The main finding is the significant and independent association between the *ADRB1* rs1801252 G (Gly49) allele and increased susceptibility to POAG across allelic, dominant, and additive genetic models, even after adjustment for age and sex and correction for multiple testing. In contrast, *ADRB2* rs1042714 showed no significant association with POAG risk. Haplotype analysis further suggested that the combined *ADRB2* C–*ADRB1* G haplotype may increase disease susceptibility. Importantly, neither polymorphism was associated with IOP or CDR, suggesting that these variants may primarily influence disease susceptibility rather than clinical severity.

The association between *ADRB1* rs1801252 and POAG susceptibility in our Saudi cohort is biologically plausible given the widespread expression of β-adrenergic receptor signaling systems in ocular tissues. The ciliary body epithelium, responsible for aqueous humor secretion, is rich in β-adrenergic receptors whose activation via the sympathetic nervous system regulates cAMP-dependent mechanisms controlling fluid secretion [9,13,23]. The *ADRB1* rs1801252 (Ser49Gly) is a non-synonymous variant that alters receptor downregulation and desensitization dynamics [17]. The G allele (Gly49) variant has been associated with enhanced agonist-promoted receptor downregulation in multiple tissues [18,19], may alter β1-receptor signaling profile in ocular tissues [24].

Although β-adrenergic signaling is classically linked to aqueous humor production and IOP regulation, our genotype-phenotype analysis did not identify significant differences in IOP across *ADRB1* genotypes, suggesting that the observed association may involve non-IOP-dependent mechanisms. β1-adrenergic signaling contributes to microvascular tone and blood-flow autoregulation at the optic nerve head and retrobulbar circulation [25,26,27]. The enhanced agonist-induced downregulation associated with the Gly49 variant [18] may potentially impair vascular responsiveness, resulting in chronic perfusion instability, localized ischemia, and laminar hypoxia that might contribute to RGC injury without sustained IOP elevation [28]. In addition, altered β1-adrenergic signaling may plausibly influence intracellular calcium-dependent stress signaling pathways, including Ca^2+^/calmodulin-dependent protein kinase II, thereby increasing susceptibility to mitochondrial dysfunction and apoptotic responses implicated in glaucomatous neurodegeneration [29,30,31]. Collectively, these mechanisms suggest that *ADRB1* rs1801252 may increase POAG susceptibility by enhancing neurovascular vulnerability and reducing retinal ganglion cell resilience through IOP-independent pathways; however, dedicated functional studies are needed to validate these mechanisms in ocular models.

Our findings differ from previous studies involving other *ADRB* gene polymorphisms. Inagaki et al. reported that *ADRB1* rs1801253 and *ADRB2* variants were associated with glaucoma-related traits such as age at diagnosis and IOP but not overall open-angle glaucoma susceptibility, except in normal-tension glaucoma [13]. While rs1801253 and rs1801252 affect different ADRB1 domains, both support the broader concept that *ADRB1* functional variation influences glaucoma biology. Our findings extend this concept by identifying rs1801252 as a susceptibility marker in the Saudi Middle Eastern population.

In contrast, *ADRB2* rs1042714 showed no association with POAG risk. This differs from reports in Indian cohorts but aligns with findings from Japanese and African ancestry studies [13,14,22]. These findings suggest that rs1042714 (Gln27Glu) variant may require specific environmental or epistatic contexts to manifest an effect, which may not be present in this population. Such discrepancies likely reflect ancestry-specific allele frequencies, linkage disequilibrium structures, or gene-environment interactions [32,33]. These observations reinforce the substantial genetic heterogeneity underlying POAG and highlight the importance of population-specific studies [7].

The modest association of the C–G combined allelic profile suggests a plausible cumulative effect of adrenergic pathway variants on POAG susceptibility. Although these loci are not in LD and formal epistatic interaction was not tested, combined variation across *ADRB1* and *ADRB2* may influence overall adrenergic signaling balance and susceptibility to glaucomatous neurodegeneration. However, larger studies are required to validate the cumulative effect observation.

The absence of association with IOP and CDR suggests that *ADRB1* rs1801252 may influence early susceptibility to POAG rather than disease severity after onset. Disease progression in established glaucoma is likely governed by multiple additional factors, including mitochondrial dysfunction, oxidative stress, neuroinflammation, extracellular matrix remodeling, and retinal ganglion cell resilience [3]. Thus, *ADRB1* variation may represent one component of a broader susceptibility framework rather than a determinant of progression.

Beyond their functional roles in ocular physiology, both *ADRB1* rs1801252 and *ADRB2* rs1042714 have been widely cataloged in public genetic repositories such as dbSNP and ClinVar due to their broader pleiotropic implications in systemic traits. For instance, the *ADRB1* rs1801252 (Gly49) variant has been independently documented in association with cardiovascular alterations, including a reduced resting heart rate and modulated myocardial responses [34,35]. Concurrently, the *ADRB2* rs1042714 (Glu27) variant has been extensively investigated regarding metabolic traits, showing notable links to obesity risk and altered lipolysis regulation across various populations [35,36,37]. Although these systemic comorbidities, such as cardiovascular status and metabolic profiles, could theoretically intersect with ocular vascular and optic nerve perfusion dynamics relevant to age-related POAG pathogenesis, our current cohort focused strictly on primary ophthalmic evaluations. Future prospective investigations integrating detailed systemic phenotypic profiling with genomic data will be valuable to evaluate the precise degree of overlapping pleiotropic risk between these cardiovascular/metabolic traits and POAG susceptibility in the Saudi population.

Limitations: This study has certain limitations, and the results need cautious interpretation. Although the observed *ADRB1* association remained statistically significant after adjustment for covariates, the modest sample size may limit statistical power for detecting smaller genotype effects, particularly for *ADRB2*. Considering the minor allele frequencies observed in our study, the estimated statistical power to detect an odds ratio (OR) of 2.0 at an alpha level of 5% was 77.7% (0.78) per allele for the *ADRB1* (rs1801252) polymorphism and 99.2% (0.99) per allele for the *ADRB2* (rs1042714) polymorphism. However, to fully substantiate our findings and rule out subtle effect sizes (OR of 1.5 or less) commonly observed in genetic association studies, independent replication in larger multicenter Arab cohorts is warranted. This power limitation may account for the lack of statistically significant associations observed with the rs1042714 polymorphism in POAG cases and during the clinical marker sub-analysis. In addition, the potential contributing effects of systemic or metabolic comorbidities, alongside β-blocker use and *ADRB* gene variants on POAG risk and IOP regulation remain to be investigated. While the lack of association between genotype and clinical phenotypes points toward pressure-independent pathways, our study lacks expression data and direct mechanistic laboratory evidence to establish the exact downstream signaling cascades or cellular changes occurring in human ocular tissues. The retrospective design precluded longitudinal assessment of disease progression or therapeutic response. Nevertheless, this study represents the first genetic analysis of these functional adrenergic receptor polymorphisms in a Middle Eastern population, which is underrepresented in ophthalmic genomics [7,38].

## 4. Materials and Methods

### 4.1. Study Design and Recruitment

A retrospective and exploratory case–control study was conducted. The study adhered to the principles outlined in the Declaration of Helsinki for human research and was approved by the Institutional Review Board at King Saud University College of Medicine, Riyadh, Saudi Arabia. Written informed consent was obtained from all study participants prior to enrollment. Study participants were recruited from the King Abdulaziz University Hospital, Riyadh, Saudi Arabia. All clinical examinations and phenotyping were performed by trained glaucoma specialists, with diagnoses based on the European Glaucoma Society guidelines [39].

### 4.2. Study Population

POAG Cases (*n* = 167): All patients underwent a comprehensive standardized ophthalmic examination including Goldmann applanation tonometry (mounted at the slit lamp), gonioscopy to assess anterior chamber angles, dilated pupil fundoscopy, and automated visual field analysis using a Humphrey Field Analyzer II (Carl Zeiss Meditec, Inc., Dublin, CA, USA) with a 24–2 full threshold program. Patients met the following inclusion criteria: (1) presence of glaucomatous optic neuropathy defined as loss of neuroretinal rim with a vertical CDR ≥0.7 or inter-eye CDR asymmetry ≥0.2, and/or optic disc notching attributable to glaucoma; (2) corresponding visual field abnormalities typical of glaucoma, including nasal step defects, arcuate or paracentral scotomata, or generalized tunnel vision; (3) bilaterally open anterior chamber angles on gonioscopy; (4) adult-onset disease (age ≥18 years); (5) IOP ≥21 mmHg in one or both eyes prior to glaucoma treatment initiation; and (6) no evidence of secondary causes of glaucomatous optic neuropathy, thereby excluding exfoliative, angle-closure, pigmentary, post-traumatic, infectious, inflammatory (e.g., uveitis), post-surgical, or medication-induced (e.g., corticosteroid-related) glaucoma.

Controls (*n* = 251): Healthy non-glaucomatous individuals of Saudi ethnicity were recruited from ophthalmology screening clinics and met the following criteria: (1) age ≥40 years; (2) normal IOP (<21 mmHg) without pharmacological treatment; (3) open anterior chamber angles on gonioscopy; (4) healthy optic disc appearance with CDR <0.5; (5) no clinical evidence of glaucoma or other ocular pathology on comprehensive examination; and (6) negative family history of glaucoma.

### 4.3. DNA Extraction and Genotyping

DNA was extracted from peripheral blood samples collected in EDTA tubes using standard protocols. The *ADRB1* rs1801252 [A>G] and *ADRB2* rs1042714 [C>G] polymorphisms were genotyped using commercially available TaqMan assays (Applied Biosystems, Inc., Foster City, CA, USA: C_8898508_10 and C_2084765_20, respectively; Catalog number: 4351379). Genotyping was performed using an ABI 7500 Real-Time PCR System (Applied Biosystems, Inc., Foster City, CA, USA) under manufacturer-recommended amplification conditions. Each PCR reaction was performed in a total volume of 25 μL, containing 1× TaqMan Genotyping Master Mix (Applied Biosystems, Inc., Foster City, CA, USA), 1× SNP Genotyping Assay Mix, and 20 ng genomic DNA. Each 96-well plate included two no-template negative controls. Genotyping was performed using automated 2-color allele discrimination software (Applied Biosystems 7500 RealTime PCR System software v2.3) with fluorescence analysis visualized in a two-dimensional scatter plot.

### 4.4. Statistical Analysis

Genotyping call rates were calculated for each single nucleotide polymorphism (SNP). Minor allele frequency was determined, and HWE in the control group was assessed using Fisher’s Exact test. Continuous variables (age) were expressed as mean ± standard deviation (SD) and compared between POAG cases and controls using the Mann–Whitney U-test (non-parametric) after testing for normality using the Shapiro–Wilk test. Categorical variables (gender) were compared using Pearson’s Chi-square test.

Genetic Association Analysis: Association between *ADRB1* (rs1801252) and *ADRB2* (rs1042714) polymorphisms and POAG risk was evaluated using both allelic and genotypic models. Allelic association and genotype associations under co-dominant, dominant, recessive, and additive models were analyzed using Pearson Chi-square test or Fisher’s Exact Test (when expected counts <5) to determine OR, 95% CI, and *p*-values. To evaluate the additive genetic effect (dose–response trend), a logistic regression model was employed, treating genotype as a continuous variable (0, 1, or 2 copies of the minor allele). Multivariate logistic regression was performed to determine if observed associations were independent of potential confounders; POAG status was the dependent variable, SNP genotype was the independent variable, and age (continuous) and gender (male = 1, female = 0) were included as covariates for adjustment.

Multiple Testing Correction: A two-tailed *p*-value < 0.05 was initially considered statistically significant. To account for multiple testing of 2 SNPs, a Bonferroni-corrected significance threshold of α = 0.025 was applied.

Combined Allelic Analysis: LD between the studied polymorphisms was assessed using the r^2^ statistic. Combined allelic frequencies of 2 SNPs were estimated using SNPStats. Differences in haplotype distribution between POAG cases and healthy controls were evaluated using Fisher’s Exact Test. OR and 95% CI were calculated to determine risk associated with specific genetic profiles.

Genotype-Phenotype Correlation Analysis: Clinical parameters including IOP and CDR were evaluated exclusively within the POAG patient cohort. Normality of clinical data distribution was assessed using the Shapiro–Wilk test. Given that both IOP and CDR exhibited significant deviations from normal distribution (*p* < 0.001 for both), the non-parametric Kruskal–Wallis H-test was used to compare median values of phenotypic traits across the three genotype groups (homozygous major, heterozygous, and homozygous minor) for both *ADRB1* (rs1801252) and *ADRB2* (rs1042714). Quantitative clinical data are presented in text as mean ± SD for clinical relevance.

Statistical Software: Statistical analyses were performed using IBM SPSS v21.0 (IBM Corp., Armonk, NY, USA) and SNPStats (https://www.snpstats.net/start.htm; accessed on 20 July 2026) online tools. Odds ratios with 95% confidence intervals from logistic regression models were visualized using a forest plot constructed in R (ggplot2; version 4.0.3). Power analysis was performed using the PS Power and Sample Size Calculations program (version 3.1.2).

## 5. Conclusions

In conclusion, the *ADRB1* rs1801252 (Gly49) variant was independently associated with significantly increased POAG susceptibility in this Saudi cohort, whereas *ADRB2* rs1042714 showed no overall association with disease risk. These findings add to the understanding of the genetic variations linked to POAG in Middle Eastern populations and suggest a direction for future functional and pharmacogenetic studies investigating the role of adrenergic receptor genetics in glaucoma. However, these findings are preliminary and population-specific, requiring validation in larger, independent cohorts. In addition, given the lack of functional experiments, further laboratory studies are needed to determine whether these variants influence disease pathogenesis through pressure-independent pathways.

## Figures and Tables

**Table 1:** Demographic and Clinical Characteristics of the Study Participants.

Characteristic	POAG Cases (*n* = 167)	Controls (*n* = 251)	*p*-Value
Age (years), Mean ± SD	61.3 ± 10.5	59.9 ± 7.8	0.046
Gender (Male/Female), *n*	94/73	136/115	0.747
Male (%)	56.3	54.2	—
Genotyping Call Rate (%)	97.6	99.2	—
HWE *p*-value rs1801252	—	0.092	—
HWE *p*-value rs1042714	—	0.220	—

HWE, Hardy–Weinberg Equilibrium.

**Table 2:** Allelic and Genotype Associations of *ADRB1* (rs1801252) and *ADRB2* (rs1042714) with POAG Susceptibility.

SNP	Model	Case vs. (*n*)/Control (*n*)	OR (95% CI)	Unadjusted *p*	Adjusted *p* ^†^
*ADRB1* (rs1801252)	Allele (A vs. G)	46/280 vs. 34/464	2.24 (1.40–3.58)	0.007	0.005
	Co-dominant	A/A vs. A/G vs. G/G	—	0.011	0.026
	Dominant ((A/G + G/G) vs. A/A)	39/124 vs. 31/218	2.21 (1.31–3.72)	0.003	0.007
	Recessive (G/G vs. A/A + A/G)	7/156 vs. 3/246	3.68 (0.94–14.44)	0.056	0.098
	Additive (per G-allele)	—	2.02 (1.29–3.14)	0.002	0.005
*ADRB2* (rs1042714)	Allele (C vs. G)	83/243 vs. 116/382	1.12 (0.81–1.56)	0.506	0.433
	Co-dominant	C/C vs. C/G vs. G/G	—	0.776	0.691
	Dominant ((C/G + G/G) vs. C/C)	69/94 vs. 99/150	1.11 (0.74–1.66)	0.610	0.588
	Recessive (G/G vs. C/C + C/G)	14/149 vs. 17/232	1.28 (0.61–2.68)	0.568	0.383
	Additive (per G-allele)	—	1.11 (0.82–1.52)	0.497	0.433

OR (95% CI), odds ratio (95% confidence interval); ^†^ Adjusted for age and gender. Significance after Bonferroni correction (*p* < 0.025).

**Table 3:** Combined Two-Locus Allelic Frequency Analysis of rs1042714 (*ADRB2*) and rs1801252 (*ADRB1*).

Allelic Combination (rs1042714–rs1801252)	Case Frequency	Control Frequency	Odds Ratio (95% CI)	*p*-Value	Significance
C–A (Major–Major)	0.664	0.721	1.00	-	Reference
C–G (Major–Minor)	0.082	0.045	1.91 (1.06–3.44)	0.033	Increased risk
G–A (Minor–Major)	0.211	0.213	0.99 (0.70–1.40)	1.000	Neutral
G–G (Minor–Minor)	0.044	0.022	2.02 (0.90–4.55)	0.096	Potential risk

Statistically significant at *p* < 0.05.

## Data Availability

The data supporting the conclusions of this article are all presented within the report.

## Supplementary Materials

The following supporting information can be downloaded at https://www.mdpi.com/article/10.3390/ijms27156668/s1.
