# Supplementary material for: Association of Functional ADRB1 (rs1801252) and ADRB2 (rs1042714) Polymorphisms with Primary Open-Angle Glaucoma in a Saudi Cohort
Source: Int J Mol Sci. 2026 Jul 26;27(15):6668. doi: 10.3390/ijms27156668 (PMC13466471; doi:10.3390/ijms27156668)
Supplement: Supplementary file 1 [file ijms-27-06668-s001.zip › ijms-4453725-supplementary.pdf]

Supplementary Table S1. Intraocular pressure and cup-to-disc ratio according to combined *ADRB2* (rs1042714) and *ADRB1* (rs1801252) genotype profiles in patients with POAG

**Combined genotype profile**	**Count**	**IOP, mean ± SD**	**IOP, median (IQR)**	**CDR, mean ± SD**	**CDR, median (IQR)**
C/C-A/A	71	21.3 ± 6.7	20.0 (17.0-25.0)	0.78 ± 0.16	0.80 (0.65-0.90)
C/G-A/A	42	21.4 ± 8.6	20.0 (15.0-26.0)	0.78 ± 0.17	0.80 (0.60-0.90)
G/G-A/A	12	18.1 ± 3.4	18.5 (15.8-20.2)	0.83 ± 0.14	0.80 (0.70-0.96)
C/C-A/G	22	24.7 ± 9.9	22.0 (17.2-28.8)	0.82 ± 0.18	0.88 (0.70-1.00)
C/G-A/G	9	19.6 ± 4.5	19.0 (16.0-20.0)	0.73 ± 0.27	0.80 (0.60-0.95)
G/G-A/G	1	26.0	26.0	1.00	1.00
C/C-G/G	1	26.0	26.0	0.65	0.65
C/G-G/G	4	17.0 ± 2.8	17.0 (16.0-18.0)	0.80 ± 0.07	0.80 (0.78-0.82)
G/G-G/G	1	28.0	28.0	0.60	0.60
**Global comparison**		--	p = 0.299	--	p = 0.532

Values are presented as Mean ± SD and Median (Interquartile Range, IQR). Global group comparisons were conducted using the non-parametric Kruskal–Wallis test. Statistical estimates for low-frequency combined profiles ($n \le 4$) should be interpreted cautiously due to small subgroup sample sizes. Post-hoc pairwise testing was not conducted because global comparisons were not statistically significant. IOP = Intraocular Pressure; CDR = Cup-to-Disc Ratio; SD = Standard Deviation.
